# Update on Disease-Modifying Pharmacological Treatments for Frontotemporal Dementia (FTD): A Scoping Review of Registered Trials

**DOI:** 10.3390/neurosci6040114

**Published:** 2025-11-13

**Authors:** Patrick Bartoshyk, Rónán O’Caoimh

**Affiliations:** 1 School of Medicine, Faculty of Education & Health Services, University of Limerick, Garraun, Castletroy, Co., V94 T9PX Limerick, Ireland; 22183795@studentmail.ul.ie; 2 Affiliation Health Research Board Clinical Research Facility, University College Cork, Mercy University Hospital, T12 WE28 Cork City, Ireland

**Keywords:** scoping review, frontotemporal dementia, interventional research, familial, sporadic, progranulin, disease-modifying therapies

## Abstract

Frontotemporal dementia (FTD) represents a cluster of adult-onset neurodegenerative diseases resulting from a combination of genetic and epigenetic factors. Currently, treatment is symptomatic and there are no licensed disease-modifying therapies available. The aim of this review was to provide an overview of ongoing or recently completed clinical studies targeting disease modification in FTD. A structured search of interventional trials of pharmacological compounds was conducted on three clinical trial registries (National Library of Medicine Clinical Trials, European Union Clinical Trials, and the Australian New Zealand Clinical Trials registries) up to September 2025. Twelve interventional trials were found. Half targeted autosomal-dominant progranulin (GRN) mutations (*n* = 6) and half examined therapies targeting neuroinflammatory-induced sporadic FTD (*n* = 6). The interim results of the early-phase (1/2) randomized controlled trials (RCTs), comprising three ongoing gene replacement studies (PROCLAIM, ASPIRE-FTD, upliFT-D) and one immune-modulating monoclonal antibody (INFRONT, now in phase 3)—all targeting the FTD-GRN mutation—show safety, tolerability, and effectiveness in restoring progranulin levels. Two recently completed phase 2 RCTs for sporadic FTD targeting neuroinflammation, the PEA-FTD and C9orf72 ALS/FTD trials, show disease-modifying potential. While interim results from six trials suggest clear mechanistic efficacy, prospective high-quality later-phase RCTs are required to ascertain long-term clinical efficacy. Since familial FTD encompasses less than half of the people with this disease, it is important to continue exploring the underlying pathophysiology, neuroimmunology, and treatment of epigenetic-induced sporadic FTD.

## 1. Introduction

Frontotemporal dementia (FTD) represents a group of adult-onset neurodegenerative diseases resulting from a combination of genetic and epigenetic, environmentally induced factors. It is the most common subtype of dementia in people under the age of 60, with an estimated point prevalence of 15 to 22 per 100,000 of the population [1,2]. The prognosis is variable, with death occurring on average between six and nine years after symptom onset [3]. The neuropathology is such that the frontal and temporal cortices of the brain, one or both, undergo selective degeneration, leading to the cognitive, behavioral, and language disturbances that occur as the disease advances [4]. Two basic phenotypes of FTD have been identified, the behavioral variant (bvFTD) and primary progressive aphasia (PPA), which is further categorized into three variants associated with language dysfunction [5]. These are the non-fluent variant (nfvPPA), semantic variant (svPPA), and logopenic variant (lvPPA) [4,6]. The associated symptoms are speech apraxia, reduced single-word comprehension, and difficulty finding words or slow speech, respectively [7]. Behavioral variant is more common, occurring in 50–70% of patients, and with an average age onset in the late fifties [4,8]. Clinical diagnosis is based on the presence of three of six features, which include apathy, disinhibition, cognitive decline, stereotypical demeanor, overeating, and loss of empathy [4]. Associated corticobasal degeneration results in concomitant motor dysfunction in about fifteen percent of these cases [6,8]. When motor neuron involvement occurs, the survival time averages about two years [8].

Autosomal-dominant inheritance is found in up to fifty percent of people with FTD [4]. Disease-associated mutation has been identified in three main genetic groups, comprising progranulin (GRN) on chromosome 17q21, C9orf72 on chromosome 9 open reading frame 72, and microtubule-associated protein tau (MAPT) [9,10]. The C9orf72 hexanucleotide repeat expansion represents a crucial genetic link between FTD and amyotrophic lateral sclerosis (ALS), two disorders now recognized as part of a clinico-pathological spectrum [11]. Individuals carrying C9orf72 mutations may present with either isolated FTD, ALS, or a mixed ALS–FTD phenotype, reflecting shared molecular mechanisms of neurodegeneration such as TDP-43 proteinopathy [11]. In clinical trials that include patients with both ALS and FTD, recruitment criteria typically depend on the presence of a confirmed pathogenic testing and or diagnostic features of FTD or ALS. Endpoints may differ slightly between groups, with motor function outcomes often prioritized in ALS cohorts and cognitive and behavioral measures emphasized for FTD participants, although shared biomarkers, such as neurofilament light chain (NfL) levels or imaging markers, are increasingly being used to assess treatment response across both conditions [12,13].

Studies have shown the type of mutation influences variant type, disease onset, symptomology, and progression [3,10]. For example, Moore et al., in a study of 3403 symptomatic individuals with FTD, determined that C9orf72 mutation was the most common cause of genetically acquired FTD, whereby MAPT and GRN mutation resulted in disease onset and death in the youngest and oldest age category, respectively [3]. GRN FTD is associated with a diverse age of onset and wide-ranging phenotypic heterogeneity and occurs in about twenty percent of familial FTD [4,14]. People with this specific mutation most often develop bvFTD or nfvPPA [4,15]. The GRN gene directs the production of the progranulin glycoprotein, essential for cell growth, division, and survival of nerve cells [15]. When GRN mutation occurs, the gene loses functionality as sortilin, a protein receptor that is essential to the regulation of progranulin cellular transport and availability, is disrupted [16]. The result is impaired lysosomal function and subsequent neurodegeneration. Progranulin protein production is inhibited to about half of what is normal, causing accumulation and aggregation of transactive response DNA binding protein of 43 kDa (TDP-43) and subsequent cellular dysfunction, neuroinflammation, and neural cell death in the frontal and temporal lobes of the brain [17]. Aside from genetic inheritance, there is increasing evidence of multifactorial epigenetic causation (i.e., environmental influences such as chemical exposure, diet, certain medications, tobacco, or alcohol) in the development of sporadic FTD [18]. This occurs over time and examples include neuroinflammation, autoimmune diseases, and head trauma [4,19]. The pathogenesis is such that biochemical cellular pathways are altered, and while the DNA sequence remains unaffected, changes in gene expression and chromatin structure can occur [18]. The result is DNA methylation, histone modification, and altered RNA processing, all of which can affect neurodegeneration [20]. Pathologically, in both familial and sporadic FTD, three main protein biomarkers have been identified: TDP–43, fused in sarcoma (FUS), and tau (structural proteins predominantly found supporting the stability of cerebral axonal microtubules) [21,22].

Current research in finding a treatment to delay, modify, or cure FTD consists primarily of gene therapy and other pharmacotherapy approaches towards the two differing clinical spectrums of autosomal-dominant mutation and sporadic epigenetic-induced neuroinflammation (with and without gene mutation). Genetically triggered therapies have a strategic advantage in targeting specific disease-associated MAPT, C9orf72, and/or GRN mutations, and they allow for early intervention, which could improve outcomes [14]. While it may be possible that certain chemical compounds can reverse epigenetic modifications, genetic mutations may be reversible, and therefore, interventional trials using gene replacement therapy hold promise. Currently, there are no licensed, regulated treatments to stop, delay, or reverse this disease. Medical management is almost exclusively symptom-based, with no evidence that pharmacological treatments used in other dementia subtypes, such as cholinesterase inhibitors or memantine-targeting glutamate neurotransmitters, are effective [23]. While most research is directed towards understanding the pathophysiology and approaches to better manage symptoms, emerging and active trials are underway examining the potential for disease-modifying treatments for FTD. Studies targeting neuroinflammation, hyperphosphorylated tau (monoclonal antibodies and anti-tau vaccination), and gene (viral vector) modification have shown no positive results to date. Yet, preliminary studies, some of which have advanced to phase 3 trials, suggest pharmacological approaches to target specific mutations in restoring progranulin levels have potential. Studies targeting tau to treat sporadic FTD have shown less promise due to the challenges in identifying the underlying pathological biomarkers. Other studies examining enzymes such as sodium selenate-activated protein phosphatase 2 A (PP2A), in tau dephosphorylation, have shown safety and tolerability, but efficacy is yet to be demonstrated [24].

There has been much progress in the field of neurodegeneration over recent years, culminating in the development of disease-modifying therapies in Alzheimer’s disease, which have now been licensed in multiple regulatory jurisdictions. Despite this, little is known about the potential for disease modification in FTD. To date, few papers have examined the current literature and evaluated ongoing studies, with most reviews instead providing a broad overview of available therapeutic strategies combined with expert opinion. These have found that the evidence for the therapeutic benefit of pharmacological agents in FTD is limited and that studies primarily focus on familial forms with identified mutations rather than sporadic disease. For example, Buccellato et al. identified trials that specifically targeted genetic cases in a recently published review of therapeutic approaches for FTD-GRN and FTD-C9orf72 mutations [25]. Their review concluded that treatments designed to treat FTD-GRN by restoring progranulin levels are the most commonplace and potentially viable approach undertaken to date [25]. The need to further explore the underlying pathophysiology to generate studies targeting tau or TDP-43 for sporadic FTD was identified as an essential step aimed at this population [25].

### Aims and Objectives

The overarching aim of this targeted scoping review is to update the evidence for disease-modifying medications by examining recently completed or ongoing clinical trials of pharmacological compounds in FTD, directed towards either familial and or sporadic FTD. Specific aims include identifying recent or current clinical trials registered between January 2019 and September 2025 that investigate pharmacological agents intended to modify the disease course of FTD. This paper also seeks to describe the types of interventions under investigation, their proposed mechanisms of action, and whether they target familial or sporadic forms of FTD, including any stratification by genetic mutation (e.g., GRN, MAPT, C9orf72). The review will also examine the development phase and objectives of each trial, geographic and institutional trends in trial activity, and the common inclusion criteria, outcome measures, and study designs employed.

## 2. Materials and Methods

This scoping review has been conducted in accordance with the Preferred Reporting Items for Systematic reviews and Meta-Analyses extension for Scoping Reviews (PRISMA-ScR) (checklist provided in the Supplementary Material). A detailed protocol was published for this review on Open Science Framework (https://doi.org/10.17605/OSF.IO/SM5UV accessed on 2 November 2025). A brief summary of the search strategy is outlined below.

### 2.1. Search Strategy

A search of current (active) or recently completed interventional studies for disease-modifying treatments of FTD was completed using three primary portal registries of the World Health Organization International Clinical Trials Registry Platform (ICTRP). The search was performed by the lead investigator (P.B). The registries searched included the following: the United States of America’s (USA) National Library of Medicine (NIH) Clinical Trials Registry, which incorporates trials from over 200 countries worldwide and is available at https://clinicaltrials.gov/; the European Union (EU) Clinical Trials Registry (EUCTR), available at https://www.clinicaltrialsregister.eu/; and the Australian New Zealand Clinical Trials Registry (ANZCTR), available at https://www.anzctr.org.au/.

The search was conducted in February of 2025 and updated in August 2025. Filters included the terms ‘frontotemporal dementia’, ‘treatment’, and ‘not yet recruiting or recruiting or active or completed’ and allowed studies including adults aged 18+ years of age. These are presented in Appendix A.

### 2.2. Eligibility Criteria for the Scoping Review

Available phase 1, 2, 3, and 4 interventional studies of pharmacological agents were examined. Only studies in English were considered and those registered on or after 1 January 2019 until 1 September 2025 were included. Studies were limited to protocols and articles, and we excluded abstract publications, book chapters, reviews, and working documents. Duplicates, those that had moved to a higher phase of study, and those that focused exclusively on symptom management rather than disease modification were excluded. A PRISMA flow diagram was produced to summarize the study selection. The PICOS framework was adapted to structure the search strategy and eligibility criteria; see Table 1.

### 2.3. Study Selection and Data Extraction

Study citations were copied to a Microsoft Excel (version 2505) spreadsheet and were screened by two reviewers (PB and RO’C). Disagreements were settled by consensus. Most ineligible study types (e.g., book chapters and working documents) could be identified from details in the registries and could thus be excluded at the title screening stage. To validate the search strategy, a set of known relevant clinical trials investigating disease-modifying treatments for frontotemporal dementia was identified in advance. The search was then tested to ensure these benchmark studies were successfully retrieved. If any were missing, the strategy was reviewed and adjusted by refining keywords, filters, or registry settings. This iterative process helped ensure the search was sensitive enough to capture all relevant trials and was documented to support transparency and reproducibility in the review process. The methodological quality and risk of bias for all included clinical trials were independently assessed by the two reviewers using the Oxford Quality Scoring System, also known as the Jadad Scale [26], a validated five-point system used primarily for randomized controlled trials, scoring studies based on three criteria, i.e., randomization (2 points awarded for a clearly randomized process), blinding (2 points awarded for double-blinding), and withdrawals (1 point), where low-quality studies score 0–1 points and higher-quality studies 4–5 points.

## 3. Results

**Figure 1:**
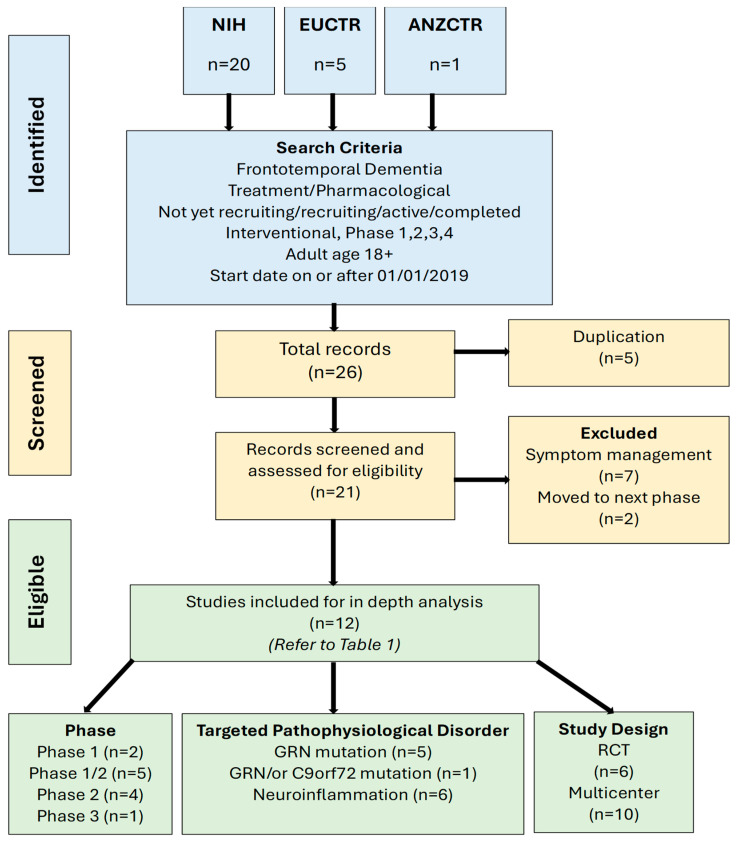
Flow diagram for the structured search and review of studies on FTD disease-modifying therapy.

The search results found 26 potential trials for inclusion. Based on the title and abstract, disease-modifying clinical trials to manage symptoms (*n* = 7), duplicate studies (*n* = 5) that were listed in both the NIH and EU registry, and trials that had advanced to the next phase (*n* = 2) were excluded, leaving 12 results. The relevant studies were retrieved for detailed evaluation. The selection of suitable clinical trials is presented in Figure 1.

Half of the studies target autosomal-dominant *GRN* mutations (*n* = 6), with the other half examining therapies targeting neuroinflammation (*n* = 6). The numbers of participants targeted for recruitment were small, ranging from 6 to 120. Of the studies completed (*n* = 2) and studies achieving target enrollment (*n* = 3), the a priori target was met or exceeded in four and is unknown in the fifth. Almost half of the trials (*n* = 5) included participants from 18 years of age. The upper age limit ranged from 75+ to no upper age limit, though most applied 85+ as the cut-off for recruitment. In all, six were randomized controlled trials (RCTs), and all of these were multi-center studies.

Table 2 presents the studies currently underway using diverse therapies, which include adeno-associated viral (AAV) vectors, protein transfer antibody vehicles, immunotherapy, and pharmacotherapies that counteract neuroinflammation. Most (*n* = 11) were phase 1 or 2 studies, with one single phase 3 trial found (NCT04374136). Half (*n* = 6) of the studies target FTD-*GRN* mutations (NCT04747431, NCT0606480, NCT04408625, NCT05262023, NCT04374136, NCT06705192). The remaining studies (*n* = 6) examine compounds that modulate neuroinflammation, with a focus on pharmacological therapies that counteract immune mediators and contribute to neurodegeneration and/or cell death. Of these, one study is employing stem cell replacement (NCT05315661) and targeting bvFTD, svPPA, or nfvPPA. The other five are studying oral agents rather than being deployed as infusion therapies. These were reported to either (1) inhibit the formation of oxidative species from microglia, which results in svPPA (NCT05184569); (2) modulate synaptic activity to mitigate the release of proinflammatory mediators (NCT04489017, NCT04220021) targeting bvFTD and ALS or any disease subtype on the FTD spectrum, respectively; (3) inhibit enzymes that activate transposable elements resulting in bvPPA (NCT04993755); or (4) alter post-translational modification of tau resulting in bvFTD or nfvPPA (ACTRN = 12620000236998). These studies on sporadic FTD, stratified for disease phenotypes (bvFTD, PPA, or both) and/or specific subtypes (svPPA, nfvPPA, bvPPA), are an important development, as previous trials have almost exclusively focused on familial forms with clearly identified genetic mutations.

Table 3 summarizes the monitored primary and secondary outcomes for each study. These include clinical measures of cognition and behavior, biomarker changes, treatment safety and tolerability, and patient-centered factors such as quality of life and caregiver burden.

**Figure 2:**
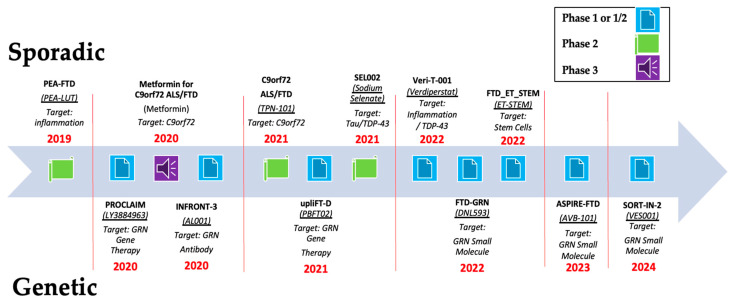
Timeline of developments in disease-modifying pharmacological treatments for frontotemporal dementia.

Each of the studies included is summarized below, and the timeline of the included studies is presented in Figure 2.

### 3.1. Studies Targeting Autosomal-Dominant GRN Mutation (n = 6)

Six clinical trials, using differing therapeutic methodologies, are currently active and designed to target the FTD-GRN mutation to restore functional levels of progranulin. The approach and preliminary findings, where available, are summarized below.

#### 3.1.1. INFRONT-3 (AL001)—NCT04374136

The phase 1, 2, and 3 INFRONT trials evaluate latozinemab (AL001), an immune-modulating recombinant human anti-human monoclonal antibody designed to block and reduce sortilin levels, thereby increasing progranulin levels, which are pathologically reduced in those with an FTD-PGRN mutation. As opposed to correcting the GRN mutation, this agent is proposed to modulate and restore progranulin levels. It is the first drug to receive a Breakthrough Therapy Designation, as well as Fast Track Designation, by the United States Food and Drug Administration (FDA) for the treatment of FTD-GRN [27]. This recombinant human monoclonal G1 antibody is designed to block sortilin interactions, thereby increasing progranulin levels and the efficacy of the progranulin gene [28]. In the phase 1 trial’s results, published in 2024, favorable safety profiles with restoration of progranulin levels were demonstrated following the administration of AL001 once every 2 weeks for a total of three doses over a 4-week period [29,30]. The seven-year phase 2 trial, designed to evaluate the safety of long-term AL001 dosing over 96 weeks, enrolled 27 people with autosomal-dominant GRN or C9orf72 mutation and is estimated to be complete in early 2026 [30]. Preliminary short-term results, released in 2021, also show a favorable safety profile, restoration of progranulin levels, renewed biomarkers including NfL, and slowing of clinical progression [30]. The phase 3 seven-year trial is a randomized, double-blind, placebo-controlled trial with a primary endpoint to slow disease progression [31]. Target enrollment of 101 (a priori target = 110) individuals across multiple centers globally was achieved in October of 2023, with patients receiving AL001 or placebo intravenously every four weeks for one to two years [27,31,32]. The phase 3 trial has excluded enrollment of people with C9orf72-associated FTD that were included in phase 2 [32]. This showed no significant impact on clinical outcomes in phase 2, despite the normalization of progranulin levels, though this may represent an important surrogate marker [32]. The results of the phase 3 trial are awaited and estimated to be reported in quarter three of 2027.

#### 3.1.2. ASPIRE-FTD (AVB-101)—NCT06064890

Preliminary results, released in 2024, from two participants in the phase 1/2 ASPIRE-FTD trial—the first human study of AVB-101, a gene therapy that also targets the GRN mutation—suggest acceptable safety and tolerability [33]. In this study, AVB-01, a DNA/RNA-based copy of the non-mutated GRN gene using an AAV vector, serotype 1 (AAV1), is infused directly into the thalamic regions of the frontal and temporal lobes [33]. The mutated gene is thereby replaced with a corrected version, which, in turn, is hypothesized to normalize progranulin levels [34]. Due to the demonstrated potential of AVB-101 to slow or halt FTD, both the FDA in the USA and the European Commission (EC) have granted orphan designation of AVB-101 for the treatment of FTD [35]. The study is ongoing, scheduled for completion in 2030, and remains in the recruitment stage (a priori target = 9).

#### 3.1.3. PROCLAIM (PR006)—NCT04408625

The PROCLAIM study also targets GRN mutation using an AAV vector, in this case, serotype 9 (AAV9), delivered with a single injection into the cisterna magna [15]. Preliminary results on 13 participants (a priori target = 30) published in 2024 demonstrate efficacy in achieving the primary endpoint of increased progranulin in all but one of the thirteen patients first enrolled [15]. The interim analysis also showed that the medium- and low-dose cohorts demonstrated progranulin levels at or above normal levels after reaching the six- and twelve-month time points, respectively [15]. As a result, PR006 received orphan drug designation for FTD from regulators in the USA and EU and fast-track designation for FTD-GRN in the USA [36]. The study is ongoing, scheduled for completion in 2030, and remains in the recruitment stage.

#### 3.1.4. UPLIFT-D (PBFT02)—NCT04747431

Randomization of 106 participants for this phase 1/2 RCT started in 2021 and is being conducted in seven centers globally. PBFT02 is a DNA/RNA-based gene replacement therapy targeting the GRN mutation, injected one time into the cisterna magna using the AAV1 vector. Preliminary results on three participants, reported in December of 2023, demonstrated safety, tolerability, and efficacy with elevated progranulin levels in the lowest dose level of three cohorts [37]. Recruitment is ongoing (a priori target = 25), and the study, originally scheduled for completion in 2027, is now pushed forward to 2031. The pharmaceutical agents in the above three trials all demonstrated acceptable safety and tolerability, with the pre-clinical and/or early-phase stages exhibiting effectiveness in restoring progranulin levels. While follow-up continues for another two to five years, it is important to note that all of these studies are preliminary, with relatively small cohorts.

#### 3.1.5. FTD-GRN (DNL593)—NCT05262023

DNL593 is a progranulin replacement therapy administered intravenously via a protein transfer antibody vehicle into the central nervous system. This RCT started in 2022 with plans to randomize 106 participants (healthy and with FTD-GRN) to DNL93 or placebo. Recruitment is ongoing, with no preliminary results reported and a study completion target of 2025. While the ASPIRE-FTD study is the only other study that assesses suicidal ideation or behavior as a primary outcome, this study uses a placebo comparator. Other primary outcomes include safety and tolerability, as well as change in physiological status (e.g., vital signs, ECG, physical/neurological findings).

#### 3.1.6. SORT-IN-2 (VES001)—NCT06705192

This phase 1a/2b single-group assignment, open-label study started in December 2024 and plans to enroll, by invitation, six asymptomatic participants with FTD-GRN. VES001, an oral, blood–brain barrier ligand of sortilin, will be administered orally in two distinct dose levels over a three-month period. The goal is to establish the safety, tolerability, and pharmacokinetics of VES001. Recruitment is ongoing and study completion is scheduled for late 2025.

### 3.2. Studies Targeting Neuroinflammation (n = 6)

Of the six clinical trials targeting sporadic neuroinflammatory FTD, detailed below, two are complete, with results recently reported; two are active, having achieved target enrollment; and the remainder (*n* = 2) are recruiting.

#### 3.2.1. PEA-FTD (Palmitoylethanolamine-Luteoline)—NCT04489017

The PEA-FTD phase 2 RCT study of people with clinically diagnosed FTD, confirmed with brain imaging, was completed in 2023 in two locations in Italy. Of the 48 participants enrolled (a priori target = 50), about half were allocated to the oral agent palmitoylethanolamine-luteoline (PEA-LUT) (*n* = 25) and the remaining to placebo (*n* = 23). PEA-LUT works by modulation of cortical oscillatory activity and GABA(B)ergic transmission. Participants with a family history of FTD underwent genetic testing and only three of those had evidence of the C9orf72 genetic mutation. The results of the study, published in 2025, demonstrated encouraging efficacy in slowing cognitive and functional decline, as measured by the Clinical Dementia Rating plus National Alzheimer’s Coordinating Center (NACC) FTLD Sum of Boxes (SB) scale [38].

#### 3.2.2. C9ORF72 ALS/FTD (TPN-101)—NCT04993755

The two-year phase 2a C9ORF72 ALS/FTD RCT of 42 participants (a priori target = 40) who were given the oral agent TPN-101, which aims to reduce neuroinflammation by inhibiting LINE-1 reverse transcriptase, concluded in 2023. The final results, released in 2024, showed safe and positive disease-modifying potential [39]. Detailed study data have not yet been published, although phase 3 planning is underway for patients with ALS.

#### 3.2.3. FTD_ET-STEM (Mesenchymal Stem Cells Preconditioned with Ethionamide)—NCT05315661

The phase 1 ET-STEM single-site trial in the Republic of Korea to evaluate the safety and tolerability of ET-STEM, launched in 2022, is scheduled for completion in 2026. Mesenchymal stem cells preconditioned with Ethionamide are shown to enhance anti-inflammatory and neuroprotective effects while promoting autophagy. They release neurotrophic factors including brain-derived neurotrophic factor and nerve growth factor. ET-STEM will be injected into the intraventricular space via an Ommaya reservoir and repeated three times at four-week intervals. While recruitment has ended, there are as yet no updates on whether the target enrollment of 12 participants was achieved and no preliminary results are available. The primary outcomes include safety, tolerability, and dose-limiting toxicity. Secondary outcomes include changes in cognitive and neuropsychiatric status and in activities of daily living. Efficacy will also be determined by CSF biomarkers.

#### 3.2.4. C9ORF72 Mutation (Metformin)—NCT04220021

The small open label, single group assignment Phase 2 trial of metformin is underway in a single site in the USA targeting C9orf72, the most commonly known FTD genetic mutation. Eighteen participants (a priori target = 16) have been enrolled and will receive the oral hypoglycemic agent metformin for 24 weeks. Metformin inhibits RNA-dependent protein kinase phosphorylation and activation and decreases Ras-related nuclear (RAN) protein levels. The study is active and not recruiting and is scheduled for completion in 2025. No interim results are available. Primary outcomes include safety and tolerability as measured by adverse events and changes in CSF RAN protein levels. The single secondary outcome will measure changes in functional ability.

#### 3.2.5. VERI-T-001 (Verdiperstat)—NCT05184569

The VERI-T-001 phase 1 RCT, initiated in 2022, is planning to enroll 64 participants with svPPA due to TDP-43 pathology. Participants will be randomized 3:1 to Verdiperstat, a molecule that inhibits myeloperoxidase—an enzyme that contributes to oxidative stress and inflammation in the brain—or placebo, administered for six months to determine safety, tolerability, and pharmacokinetics. No preliminary results or information on recruitment numbers is as yet available, and the study is scheduled for completion in 2026.

#### 3.2.6. SEL002 (Sodium Selenate)—UTN U-1111-1248-2724

This singular phase 2b RCT located on the Australia/New Zealand registry (ACTRN = 12620000236998) is studying sodium selenate as a tau-modifying agent, which works by upregulating the activity of protein phosphatase 2 in the brain, increasing the rate of tau dephosphorylation. It is scheduled for completion in 2026, and 12 participants have been enrolled (a priori target = 120). The pre-phase 1b open-label trial of 12 participants with bvFTD, published in 2022, demonstrated safety and tolerability, and while there was no evidence of change in total tau (t-tau) or phosphorylated tau (p-tau) protein levels, or positive effects on cognition or behavior, the brain atrophy rate decelerated in the majority of participants included in the trial (*n* = 7) [24].

### 3.3. Methodological Quality of Included Trials

The methodological quality of the twelve included trials, as quantified by the Jadad Scale [26], are presented in the Appendix B. This demonstrates a bimodal distribution, with scores of either 1 or 5. Six studies achieved the maximum score of 5, signifying a high level of methodological quality due to their randomized, double-blind, and placebo-controlled designs (i.e., the FTD-GRN, INFRONT-3, Veri-T-001, PEA-FTD, C9ORF72 ALS/FTDl and SEL002 trials, most of which were phase 2 or 3 RCTs). The remaining six received the lowest possible score of 1. These low-scoring studies are characteristic of and reflect phase 1 and phase 1/2 safety and dose escalation designs, which are typically non-randomized and open-label (i.e., the low scores in this latter group primarily reflect their early-phase design and not necessarily flaws in study execution).

## 4. Discussion

This scoping review identified recently completed or ongoing studies published in clinical trials registries since 2019, examining potentially disease-modifying treatments and other drug therapies designed to treat FTD. It found that half of the studies identified examined pharmacological approaches to target specific genetic chromosomal or single-gene mutations in restoring progranulin levels. Of these, only latozinemab (AL001) has advanced to a phase 3 RCT in the INFRONT trials [30,31,32], highlighting the nascency of this field of research. The INFRONT phase 1 and 2 trials restored progranulin levels to a normal range, showed favorable safety profiles, and slowed clinical progression by 47% [30]. The other progranulin replacement trials found are due for completion, with pre-clinical trials in mice showing neuronal preservation and preliminary results in humans demonstrating safety and increased cerebrospinal PRGN concentrations [40].

Another important therapeutic approach is gene and stem cell replacement. As noted, AVB-101 and PR006 in the ASPIRE-FTD and PROCLAIM phase 1/2 gene replacement trials, respectively, have already received fast-track designation due to preliminary results showing efficacy in restoring progranulin levels [33,36]. The uplift-D trial is similar to ASPIRE-FTD in that it uses the AAV1 as a vector-carrying agent. While the ET-STEM trial is still in early-phase stage 1, with a small cohort of 12 participants, its predicted ability to stimulate neural regeneration with minimal dosing would be a significant breakthrough.

Most studies were conducted in the USA and EU, suggesting limited geographic dispersion. The singular Australia/New Zealand trial (ACTRN = 12620000236998), studying sodium selenate as a tau-modifying agent, will be of particular interest due to the challenges associated with identifying the underlying pathological biomarkers of tau phosphorylation. In preliminary findings, while there was no evidence of change in t-tau or p-tau protein levels, or positive effects on cognition or behavior, the brain atrophy rate decelerated in the majority of participants included in the trial [24]. In this context, it is possible that the timing of the treatment, namely employing pharmacological therapy in the early stages of pathogenesis (brain atrophy), may increase efficacy, hence the importance of the larger cohort phase 2b RCT, which includes a control group and people in different phases of the disease.

The remaining four phase 1 and 2 studies targeting neuroinflammation that were found in this search examine oral pharmaceutical agents to target specific variants and mutations of FTD. TPN-001, in the C9orf72 ALS/FTD trial, has already shown safety and efficacy in the treatment of people with Human Immunodeficiency Virus (HIV) [41]. It is further projected that, in the presence of FTD, the loss of functional nuclear TDP-43 produces a neuroinflammatory antiviral immune response that may be counteracted by TPN-101 [41]. The results of this study show disease-modifying potential [41]. Similarly, Verdiperstat, a myeloperoxidase inhibitor administered in the Veri-T-001 trial, restricted to participants with svPPA in the absence of tau pathology, targets TDP-43 pathology by reducing oxidative stress and subsequent activation of cell-damaging microglia [42]. However, unlike the results of the TPN-001 agent, previous studies using Verdiperstat as a disease-modifying therapy for Parkinson’s disease, multiple system atrophy, and ALS, all diseases with neuroinflammatory characteristics similar to FTD, failed to show differences compared to placebo [42].

The PEA-FTD phase 2 RCT study of 48 people with bv-FTD demonstrated encouraging efficacy in slowing cognitive and functional decline [38]. Since only three participants demonstrated evidence of familial genetic mutation, it is assumed the remaining (*n* = 45) had sporadic FTD. However, long-term efficacy in halting the disease has yet to be proven in large, multicenter RCTs. Lastly, in the small phase 2 single-site metformin trial of 18 participants for the most commonly known FTD genetic mutation (C9orf72), it is hypothesized that metformin may restore mitochondrial function by inhibiting abnormal dipeptide repeat proteins in the presence of ALS and FTD [43]. Metformin is an agent commonly used for the management of diabetes and hence, its safety profile has already been established in that population. Given that this is a repurposed drug, if efficacious, its low cost and availability would make it an important cost-effective therapy.

### 4.1. Methodology and Trial Design

Across the reviewed trials, several shared themes emerged in trial design and methodology. Safety and tolerability were universally prioritized as primary endpoints, particularly in early-phase gene therapy and monoclonal antibody studies, many of which included long-term safety follow-up extending up to five years. Biomarker measurement was a central component of outcome assessment, with plasma and CSF GRN and NfL levels frequently used as pharmacodynamic markers of target engagement and neurodegeneration, respectively. Structural brain imaging, particularly volumetric MRI, was commonly employed to monitor neuroanatomical changes over time, with only a few studies incorporating novel tools like optical coherence tomography (upliFT-D; NCT04747431) and transcranial magnetic stimulation electroencephalography (PEA-FTD; NCT04489017).

Despite their central role as measurement tools in these trials, current biomarkers in FTD have notable limitations. Plasma and CSF NfL and progranulin levels are sensitive indicators of axonal injury but lack disease specificity, with similar elevations occurring in ALS and many other neurodegenerative conditions; hence, there is no ‘gold standard’ biomarker available [44,45]. Moreover, increases in progranulin levels confirm pharmacodynamic response in GRN-targeted therapies but do not necessarily correlate with clinical improvement [46]. Neuroimaging biomarkers, including volumetric MRI and emerging TDP-43 or tau-specific PET tracers, hold promise for tracking disease progression, but standardization across centers and the absence of validated ligands remain challenges [45]. Future trials should therefore integrate multimodal biomarker panels combining molecular, imaging, and digital measures to better capture treatment effects and disease trajectory.

Cognitive and functional outcomes were measured using standardized tools including the Clinical Dementia Rating (CDR) scale, Mini-Mental State Examination (MMSE), Addenbrooke’s Cognitive Examination (ACE), and broader neuropsychological batteries designed for FTD populations. Most trials prioritized safety and tolerability, particularly in early-phase studies, with long-term follow-up in gene therapy interventions. The overall sample sizes were modest (ranging from 6 to 120 participants), reflecting both the rarity of specific genetic subtypes and the early developmental stage of many interventions. Hence, this review found a bimodal distribution in trial quality, with only half of the included studies identified as high-quality designs and the other half representing low-quality, non-randomized, open-label phase 1 and 1/2 designs, which also reflects the nascent state of clinical development in FTD, particularly for novel modalities like gene therapies, where initial feasibility and safety must inherently supersede the methodological rigor of blinding and randomization.

While six of the trials were multi-center RCTs, the remainder were open-label or early-phase dose escalation studies, highlighting the field’s ongoing transition from safety-focused exploration to efficacy-driven evaluation. Importantly, trials targeting sporadic FTD remain underrepresented, underscoring the need for expanded research into non-genetic disease mechanisms and treatment strategies.

### 4.2. Strengths and Limitations

This review provides an up-to-date overview of available ongoing or recently completed trials targeting both genetic mutations and neuroinflammation by thoroughly searching three large trial registries. While this review offers a timely synthesis of emerging pharmacological trials in FTD, several limitations should be acknowledged. Firstly, despite using three major clinical trial registries, this approach inherently omits unpublished or unregistered studies and potentially excludes high-quality trials reported in other regional registries or non-English languages. This creates a risk of publication and language bias that may skew the perceived global distribution and diversity of trial efforts. Notably, many Asian, Eastern European, or South American initiatives may be underrepresented due to their absence from the included databases [47]. Second, while scoping reviews are designed to map existing research rather than appraise quality, the lack of formal risk-of-bias assessment limits the ability to evaluate the robustness and reproducibility of trial findings [48]. This said, we conducted a quality assessment using a recognised descriptive quality appraisal matrix [26] and risk-of-bias assessment may become a mechanical exercise that overlooks study context and fails to distinguish between minor and major biases or their impact on results [48]. While the reviewed studies demonstrate impressive methodological rigor within the constraints of rare disease research, several challenges limit their interpretability and translational potential. Most early-phase gene therapy and monoclonal antibody trials prioritize biomarker outcomes over functional endpoints, which, while informative for target engagement, provide limited insight into real-world clinical benefit. Mechanistically, interventions vary considerably, from progranulin restoration and neuroimmune modulation to tau dephosphorylation, yet cross-trial comparisons are complicated by differing inclusion criteria, disease subtypes, and outcome measures. Furthermore, the concentration of research in genetically defined subgroups, particularly GRN and C9orf72 mutations, may limit the generalizability of findings to the more heterogeneous sporadic FTD population. Addressing these gaps through harmonized protocols, cross-consortia collaboration, and inclusion of broader phenotypic cohorts will be key to translation. Many of the included trials were early-phase studies with open-label or single-arm designs, which, while appropriate for exploratory purposes, offer limited insight into efficacy and are vulnerable to placebo effects and selection bias [49]. 

Third, most trials identified focus on rare familial forms of FTD, particularly GRN mutations. While biologically tractable and often more homogenous, these cases represent a minority of the FTD population [50]. As such, the heavy focus on GRN-targeted therapies may not generalize to the majority of patients with sporadic FTD, who are underrepresented in current clinical research. This limits the translatability of findings to broader clinical practice, a concern echoed in the literature on neurodegenerative trial design [47]. Additionally, small sample sizes (as few as six participants in some trials) and long recruitment periods raise concerns about statistical power and generalizability [51]. Given the heterogeneity of FTD phenotypes and the underlying pathologies, adequately powered subgroup analyses are often not feasible. This risks overstating treatment effects or masking subgroup-specific responses, especially when diverse variants (bvFTD, svPPA, nfvPPA) are grouped together [50]. The reliance on biomarker surrogates (e.g., CSF or blood PGRN, NfL) as primary outcomes in several trials, while necessary in early-phase research, introduces further limitations [52]. While such biomarkers, including blood markers, indicate target engagement, their relationship with long-term clinical outcomes in FTD remains unclear [53]. Moreover, the lack of standardization across trials in terms of cognitive and functional measures reduces comparability and hinders meta-analytic synthesis across studies [54]. Finally, this review did not include a bibliometric or network analysis, which could have offered valuable insights into patterns of collaboration, geographic research hubs, and trial sponsorship, factors increasingly recognized as shaping the global research agenda in rare neurodegenerative diseases [47].

### 4.3. Future Challenges

While the initial safety and progranulin restoration data for therapies (e.g., AL001 in INFRONT3) and the GRN gene replacement studies (e.g., PROCLAIM, ASPIRE-FTD) are encouraging, their translation to routine clinical practice faces significant hurdles. Gene delivery challenges in neurodegenerative disorders necessitate specialized methods, such as intrathecal or intracisternal administration, which carry inherent risks of adverse events and require specialized infrastructure. Furthermore, as these therapies are delivered, long-term safety and immune response monitoring are paramount, particularly with AAV vectors used in gene therapies. Logistically, recruiting the necessary cohort of rare, autosomal-dominant variant carriers for late-stage trials presents a major challenge, contributing to the small sample sizes observed in this review (*n* = 6 to 120). Similarly, the extraordinary cost of developing and delivering one-time curative gene therapies raises complex ethical questions regarding equitable access. FTD drug development can and must draw lessons from related neurodegenerative fields. Specifically, the challenges in Alzheimer’s disease (AD) trials, where the failure of several β-secretase 1 (BACE1) inhibitors [55] demonstrates the risk of targeting a surrogate biomarker too late in the disease course, underscoring the necessity of early intervention in FTD. Conversely, the success of targeted antisense oligonucleotide (ASO) therapy in superoxide dismutase 1 (SOD-1) SOD1-ALS [56] provides a powerful analogue for similar trials in FTD, validating that taking a precision medicine approach for genetic FTD could have similar success. However, it also necessitates adopting the robust natural history and biomarker development frameworks pioneered in the AD and ALS fields to define appropriate clinical endpoints. The majority of FTD cases are sporadic, where epigenetic changes and environmental factors likely drive the underlying proteinopathy (primarily TDP-43). This highlights the importance of the six trials targeting neuroinflammatory-induced sporadic FTD, such as the PEA-FTD study. The rationale here is to modulate the microglial-mediated inflammatory response that contributes to neurodegeneration, moving away from protein clearance alone. Disease modification in sporadic FTD will require a strategy that addresses these non-genetic drivers. The ‘disease-modifying potential’ noted in the PEA-FTD trial is likely tied to the successful reduction in inflammatory markers or stabilization of functional decline, supporting evidence that immune modulation is a viable therapeutic avenue for the non-familial FTD population [57]. Future clinical success hinges on overcoming barriers to late-stage translation. The field urgently requires the development of validated surrogate biomarkers (e.g., NfL, specific tau PET ligands) that robustly correlate with clinical decline, moving beyond progranulin restoration alone [44]. The clinical and pathological heterogeneity of FTD necessitates a major shift toward precision trial design, utilizing multi-omics and AI-based patient stratification to select more homogeneous trial cohorts. Finally, the FTD research community must leverage its methodological advances, especially in imaging and fluid biomarkers, to influence trials for related tauopathies (e.g., corticobasal degeneration).

## 5. Conclusions

In this scoping review, we highlight scientific studies that are currently underway for the curative treatment of FTD. It presents and discusses current state-of-the-art research on treatments for people with FTD and identifies preliminary results, particularly in the treatment of familial FTD-GRN, which are in an early phase of development. While none of the studies found have sufficient follow-up to determine long-term efficacy, some positive short-term results for familial forms of FTD have been published. However, data are preliminary, studies are few, and sample sizes are small. As those with familial forms of the disease represent less than half of people with this disease, it will be important to continue exploring the underlying pathophysiology, neuroimmunology, and ultimately, treatment, of epigenetic-induced sporadic FTD. Additionally, since early diagnosis may enhance the effectiveness of therapy, this strengthens the case to proactively promote genetic testing in the offspring of those found to have inheritable FTD and in all people with the disease, regardless of presumed cause. The results also highlight a critical gap between positive biomarker normalization and the demonstration of sustained, clinically meaningful long-term efficacy in studies, suggesting that any positive trial findings may represent improvements in surrogate or biological markers but not patient-important outcomes. Prospective, high-quality later-phase RCTs are urgently needed to fully ascertain long-term efficacy, validate functional outcomes, and determine whether these therapeutic strategies can successfully alter the trajectory of FTD.

## Figures and Tables

**Table 1:** PICOS framework (adapted for scoping review).

Element	Description
Population	Adults (18+) diagnosed with frontotemporal dementia (any subtype, including bvFTD and PPA variants), either familial or sporadic
Intervention	Pharmacological agents with a stated aim of disease modification (e.g., gene therapy, anti-tau therapies, anti-inflammatory agents)
Comparator	Not applicable (scoping review includes all study types regardless of comparator use)
Outcomes	Study objectives related to disease modification, including biomarker changes, cognitive/functional endpoints, and trial phase progression
Study Design	Registered interventional clinical trials (phases 1–4) between 1 January 2019 and 1 September 2025

**Table 2:** Characteristics of clinical studies targeting disease modification in frontotemporal dementia (FTD).

Targeted Pathophysiological Disorder (Therapy Action)	Identification Number Phase Timeline	Study Name	Sponsor Study Design Study Locations	N	Age	Study Agent	Treatment Study Arms
**Autosomal-dominant *GRN* mutation** resulting in progranulin deficiency (DNA/RNA-based gene replacement therapy using adeno-associated virus (AAV1) vector)	NCT04747431 Phase1/2 Start: 2021-09 Primary Completion: 2028-08 Study Completion: 2031-08	**upliFT-D** A study of PBFT02 in patients with frontotemporal dementia and progranulin mutations (FTD-GRN) (upliFT-D)	Passage Bio Inc. (Philadelphia, PA, USA) Interventional Sequential Assignment, Multicenter, Non-Randomized, Open-Label, Single-Arm, Dose-Escalation 7 locations in USA, Portugal, Canada, Brazil	25	35–75	PBFT02	PBFT02 injected once into the cisterna magna 3 cohorts: Dose level 1 Dose level 2 Optional 3rd dose based on results of the first 2 cohorts
**Autosomal-dominant GRN mutation** resulting in progranulin deficiency DNA/RNA-based gene replacement therapy using adeno-associated virus (AAV1) vector	NCT06064890 Phase 1/2 Start: 2023-08 Primary Completion: 2030-10 Study Completion: 2030-10	**ASPIRE-FTD** A study to evaluate the safety and effect of AVB-101, a gene therapy product, in subjects with a genetic sub-type of FTD (FTD-GRN)	AviadoBio Limited Interventional Sequential Assignment, Non-Randomized, Open-Label, Ascending Dose Multicenter 13 locations in USA, Poland, Spain, Sweden, The Netherlands, UK	9	30–75	AVB-101	AVB-101 injected once directly into the thalamus 2 cohorts: Initial dose Escalated dose
**Autosomal-dominant *GRN* mutation resulting in progranulin deficiency** (DNA/RNA-based gene replacement therapy using adeno-associated virus (AAV9) vector)	NCT04408625 Phase 1/2 Start: 2020-11 Primary Completion: 2030-04 Study Completion: 2030-04	**PROCLAIM** Phase 1/2 Clinical Trial of LY3884963 in Patients with Frontotemporal Dementia with Progranulin Mutations (FTD-GRN) (PROCLAIM)	Prevail Therapeutics Interventional, Multi-Center, Open-Label Ascending Dose 11 locations in USA, Australia, Belgium, UK, France, Spain	30	30–85	LY3884963 (PR006)	LY3884963 injected once into the cisterna magna 3 cohorts: Low dose Medium dose Bridging, alternating low or medium dose
**Autosomal-dominant *GRN* mutation resulting in progranulin deficiency** (progranulin transfer antibody vehicle)	NCT05262023 Phase 1/2 Start: 2022-02 Primary Completion: 2025-11 Study Completion: 2025-11	**FTD-GRN** A Study to Evaluate the Safety, Tolerability, Pharmacokinetics, Pharmacodynamics of DNL593 in Healthy Participants and Participants with Frontotemporal Dementia (FTD-GRN)	Denali Therapeutics Inc. Interventional, Multicenter, Randomized, Placebo-Controlled, Double-Blind, Parallel Assignment 29 locations in USA, Belgium, Brazil, Spain, Columbia, Czechia, France, Italy, Turkey, Serbia, The Netherlands, Portugal, UK	106	18–80	DNL593 or Placebo	DNL 593 or placebo, single and multiple doses in two parts, comparing healthy participants to those with FTD
**Autosomal-dominant *GRN* mutation** resulting in progranulin deficiency (immunotherapy; sortilin-targeted monoclonal antibody called latozinemab)	NCT04374136 Phase 3 Start: 2020-07 Primary Completion: 2025-09 Study Completion: 2027-08	**INFRONT-3** A phase 3 study to evaluate efficacy and safety of AL001 in frontotemporal dementia (INFRONT-3)	Alector Inc. Interventional, Multicenter, Randomized, Double-Blind, Placebo-Controlled 44 locations in USA, Germany, Argentina, UK, Australia, Italy, Belgium, Spain, Canada, Greece, Turkey, Portugal, Sweden, France Switzerland, The Netherlands	110	25–85	AL001 (latozinemab) or Placebo	AL001 or placebo IV every 4 weeks for 1–2 years
**Autosomal-dominant** ***GRN* or *C9orf72* mutation** resulting in progranulin deficiency (small-molecule sortilin inhibitor)	NCT06705192 Phase 1a/2b Start: 2024-12 Primary Completion: 2025-06 Study Completion: 2025-09	**SORT-IN-2** Study in Asymptomatic *GRN*-FTD Patients to Investigate the Safety, Tolerability, Pharmacokinetics, and Pharmacodynamics of VES001 (SORT-IN-2)	Mads Kjolby Vesper Biotechnologies ApS Interventional Single-Group Assignment, Open-Label, Multiple-Dose 2 locations in The Netherlands, UK	6	18–75	VES001 (an oral, blood–brain barrier ligand of sortilin)	VES001 given orally in 2 distinct consecutive dose levels over a 3-month period Dose 1: 360 mg Dose 2: 900 mg
**Neuroinflammation** resulting in svPPA with TDP-43 pathology (small-molecule myeloperoxidase inhibitor to reduce oxidative-stress and pathologic activation of microglia, which lead to cell death)	NCT05184569 Phase 1 Start: 2022-04 Primary Completion: 2026-06 Study Completion: 2026-09	**Veri-T-001** Veri-T: A Trial of Verdiperstat in Patients With svPPA Due to TDP-43 Pathology (Veri-T-001)	Peter Ljubenkov, MD Interventional Parallel Assignment, Randomized 3:1, Double-Blind, Placebo-Controlled 5 locations in USA	64	18–85	Verdiperstat or Placebo	Verdiperstat or placebo 2 tablets twice daily for 24 weeks (total daily dose of 600 mg, following a one-week titration period of 1 tablet daily)
**Neuroinflammation** resulting in bvFTD (modulates synaptic activity to mitigate the release of proinflammatory mediators)	NCT04489017 Phase 2 Start: 2019-06 Primary Completion: 2022-12 Study Completion: 2023-06	**PEA-FTD** Palmitoylethanolamide Combined with Luteoline in Frontotemporal Dementia Patients: A Randomized Controlled Trial (PEA-FTD)	I.R.C.C.S. Fondazione Santa Lucia Interventional, Randomized, Controlled 2 locations in Italy	50	40–85	PEA-LUT or Placebo	Dietary supplement PEA-LUT or placebo oral dosage of 700 mg × 2/day for 24 weeks
**Neuroinflammation** resulting in 1 of 3 FTD subtypes: ♦ bvFTD ♦ svPPA ♦ nfvPPA (replacing damaged or lost cells with differentiated neuronal cells)	NCT05315661 Phase 1 Start: 2022-07 Primary Completion: 2026-12 Study Completion: 2026-12	**FTD_ET-STEM** Clinical Assessment on the Safety and Potential Efficacy of Mesenchymal Stem Cells Preconditioned with Ethionamide (ET-STEM) in Patients with FTD	Samsung Medical Center Interventional Single-Group Assignment, Open-Label 1 location in Republic of Korea	12	40–85	ET-STEM	ET-STEM 3 repeated doses at 4-week intervals; injected into intra-ventricular space via an Ommaya reservoir
**Neuroinflammation** C9orf72 mutation resulting in ALS/FTD (type not specified) (small molecule that stimulates synaptic plasticity and moderates the immune response)	NCT04220021 Phase 2 Start: 2020-01 Primary Completion: 2024-12 Study Completion: 2025-06	**Metformin for C9orf72 ALS/FTD** Safety and Therapeutic Potential of the FDA-approved Drug Metformin for C9orf72 ALS/FTD	University of Florida Interventional, Single-Group Assignment, Open-Label Single location in Florida, USA	18	18–80	Metformin	Metformin 500 mg with weekly escalation by 500 mg to maximal dose of 2000 mg × 24 weeks
**Neuroinflammation** C9orf72 mutation resulting in bvFTD and/or PPA (DNA/RNA-based small molecule to reduce biomarkers of neuroinflammation and neurodegeneration)	NCT04993755 Phase 2a Start: 2021-10 Primary Completion: 2023-09 Study Completion: 2023-09	**C9ORF72 ALS/FTD** A Phase 2a Study of TPN-101 in Patients with ALS and/or FTD Associated with Hexanucleotide Repeat Expansion in the C9orf72 Gene (C9ORF72 ALS/FTD)	Transposon Therapeutics Inc. Multi-Center, Randomized, Double-Blind, Placebo-Controlled Parallel-Group, 2-Arm Open-Label Treatment 19 locations in USA, Belgium, France, Germany, Spain	42	18+	TPN-101 or Placebo	TPN-101 400 mg/day × 24 weeks (double-blind) followed by TPN-101 × 24 weeks (open-label) or Placebo once daily × 24 weeks (double-blind) followed by TPN-101 × 24 weeks (open-label)
**Neuroinflammation** resulting in bvFTD or nfvPPA (alters post-translational modification of tau)	ACTRN = 12620000236998 UTN U1111-1248-2724 Phase 2 Start: 2021-08 Primary Completion: 2026-01 Study Completion: 2026-05	**SEL002** A Phase 2b Randomised Controlled Trial of Sodium Selenate as a Disease Modifying Treatment for Possible Behavioural Variant Fronto-temporal Dementia	National Health & Medical Research Council (NHMRC) of Australia Interventional Parallel Assignment, Randomized, Controlled 2 locations in Australia, New Zealand	120	35+	Sodium selenate or Placebo	Sodium selenate 15 mg orally three times per day × 52 weeks or Placebo

ALS = amyotrophic lateral sclerosis; bvFTD = behavioral variant frontotemporal dementia; GRN = Granulin; nfvPPA = non-fluent variant primary progressive aphasia; PPA = primary progressive aphasia.

**Table 3:** Data collection for clinical studies targeting disease modification in frontotemporal dementia (FTD) including outcomes, measures, and time points.

Study Name and Identifier	Primary or Secondary	Outcome	Measurement Tool	Time Points
upliFT-D NCT04747431	** Primary **	Safety and tolerability	Number of participants with treatment-related AEs, SAEs	Baseline up to 5 years (multiple visits)
		Change in nerve conduction velocity and amplitude	Conventional nerve conduction studies	
		Change in cellular and humoral response against the vector and transgene in serum	ELISpot and antibody titers against AAV1 and human progranulin	
	** Secondary **	Effect of PBFT02 on CSF and plasma PGRN levels	CSF and plasma PGRN levels	Baseline to 5 years (multiple visits)
		Effect of PBFT02 on PGRN level in CSF and plasma	Plasma and CSF NfL levels	
		Change in brain volume, white matter integrity, and cortical thickness	MRI	
		Changes in retinal thickness and retinal lipofuscin deposits as markers of disease progression	Optical coherence tomography	
		Change in FTLD disease progression	CDR plus NACC FTLD-SB Score (behavior and language domains)	
ASPIRE-FTD NCT06064890	** Primary **	Safety and tolerability	Type, number, and incidence of AEs and SAEs	Up to week 26
		Time to achieve clearance of vector genomes	Plasma and semen (males) measurements	
		Change in cognitive status	MMSE	Up to week 12
		Incidence of treatment emergent suicidal ideation or behavior	C-SSRS	26 week initial and 5-year total follow-up
		Incidence of treatment-emergent clinically significant abnormalities	Clinical examination Safety laboratory values	5-year total follow-up
		Change in brain structure	MRI	
	** Secondary **	Change from baseline in PGRN protein levels	Blood and CSF PGRN levels	26-week initial and 5-year total follow-up
		Change from baseline in NfL levels in CSF and blood	NfL levels	
		Change in cognitive and global function	CDR + NACC FTLD-SB score	5-year total follow-up period
		Change in brain volumes	3DT1 MRI scans	
		Change in AAV9 immunogenicity in blood	Level of antibodies and ELISPOT to AAV9 capsid	
		Change in AAV9 immunogenicity in CSF	Level of antibodies to AAV9 capsid	
		Change in PGRN immunogenicity in CSF	Level of antibodies to PGRN protein	
		Change in PGRN immunogenicity in blood	Level of antibodies and ELISPOT to PGRN protein	
		Caregiver Global Impression of Change (CaGI-C)	CaGI-C 7-point scale assessed by the caregiver	
		Patient Global Impression of Change (PGI-C)	PGI-C 7-point scale assessed by the patient	
		Clinical Global Impression of Change (CGI-C)	CGI-C 7-point scale assessed by the investigator	
		Assessment of various cognitive domains: language, attention/processing speed, executive function, verbal and visuospatial memory, and social cognition	Neuropsychological test battery for GRN-specific genetic disease Frontotemporal Initiative Cognitive (GENFI-Cog)	
**PROCLAIM** NCT04408625	** Primary **	Safety and tolerability	Number of AEs and SAEs AEs leading to discontinuation Sum of ARs and serious ARs, and suspected ARs/ serious ARs	5 years
		Incidence of procedure- or treatment-emergent AEs	Brain and spine MRI	
		Change in PGRN and AAV9 immunogenicity in blood	Level of antibodies and ELISPOT	Baseline and month 12
		Change in PGRN immunogenicity in CSF	CSF: cerebrospinal fluid	
		Change in AAV9, PGRN, and NfL immunogenicity in CSF	Antibody levels	
		Change in PGRN levels	PGRN blood and CSF levels	
	** Secondary **	Change in FTLD disease progression	CDR and NACC FTLD domains	Baseline and month 12
		Change in NfL levels	NfL blood and CSF levels	
**FTD-GRN** NCT05262023	** Primary **	Safety and tolerability	Treatment-emergent AEs: - Incidence, severity, seriousness - Incidence of clinically significant abnormalities in safety lab values	Up to 18 months
		Change in systolic and diastolic blood pressure, heart rate, respiratory rate, body temperature	Vital signs measurements	
		Change from baseline in electrocardiogram (ECG) results including PR, QRS, and QTcF intervals	ECG	
		Incidence of treatment-emergent clinically significant abnormalities in physical/neurological examination findings	Clinical examination	
		Change from baseline in suicidal ideation or behavior	C-SSRS (Parts B and C only)	
	** Secondary **	Pharmacokinetic analysis of DNL593 in serum	Pharmacokinetic parameter measurements: Cmax, tmax, AUC, t1/2, Accumulation ratio, trough concentration, AUC from time 0 to the end of the dosing interval	Up to 18 months
			AUC from time zero to infinity (AUC∞) of DNL593 in serum (Part A only)	Up to 84 days
		Change in DNL593 in CSF	Concentration of DNL593 in CSF and serum concentration ratio	Up to 18 months
		Percentage change in NfL	Plasma NfL levels	
**INFRONT-3** NCT04374136	** Primary **	Evaluation of efficacy of AL001 on FTLD behavior and language	CDR^®^ plus NACC FTLD-SB score	Through study completion, on average up to 96 weeks
	** Secondary **	Change in severity of disease relative to the clinician’s past experience with patients who have the same disease	Clinical Global Impression-Severity (CGI-S) score	Baseline to 96 weeks
		Improvement or worsening of disease relative to baseline	Clinical Global Impression-Improvement (CGI-I) score	
		Change in cognitive status (attention, language, visuospatial/construction index, memory)	Respectable Battery for the Assessment of Neuropsychological Status (RBANS) score	
		Change in pharmacodynamic biomarkers	MRI and blood/CSF NfL and PRGN	
		AL001 safety and tolerability	Incidence of AEs	
**SORT-IN-2** NCT06705192	** Primary **	Change from baseline in PGRN CSF and plasma levels in CSF	PGRN CSF levels (3 samples) and plasma levels (32 times)	Day 28 and day 84
	** Secondary **	CSF PK profile of VES001 following multiple oral doses	Analysis of two (2) CSF PK parameters for each of Cmax, tmax, t1/2, Vz/F, CL/F	16 weeks
		Concentration of VES001 in CSF and plasma/CSF ratio versus baseline	Analysis of three (3) CSF samples	Day 28 and day 84
		Safety and tolerability of VES001	CTCAE v 4.0 assessment of treatment-related AEs, SAEs, and Suspected Unexpected Serious Adverse Reactions (SUSARs) Clinical lab blood and urine tests 12-lead ECG Vital signs (blood pressure, heart rate, respiratory rate, body temp.) Physical/neurological clinical exam C-SSRS	16 weeks
**Veri-T-001** NCT05184569	** Primary **	Safety and tolerability of Verdiperstat	Assess AEs	24 weeks
	** Secondary **	Changes in pharmacokinetic and pharmacodynamic properties of Verdiperstat	Steady-state CSF and plasma concentrations of Verdiperstat and its metabolites Plasma myeloperoxidase (MPO) activity	24 weeks
		Changes in pharmacodynamic properties of CSF biomarkers	CSF concentrations of NfL	
		Change in brain volume and structural/functional connectivity	MRI	
		Change in cognitive function	CDR plus NACC FTLD score	
		Change in executive brain function	National Institutes of Health Executive Abilities Assessment (NIH EXAMINER)	
		Change in language function and semantic fluency	- Boston Naming Test - Delis–Kaplan Executive Function System (D-KEFS)	
		Change in language naming function	Digitalized analysis of prompted monolog and a picture description task on a mobile application	
		Change in neuropsychiatric function	NPI questionnaire	
**PEA-FTD** NCT04489017	** Primary **	Change in global disease severity	CDR FTLD-SB score	24 weeks
	** Secondary **	Change in executive functions	Frontal Assessment Battery (FAB)	24 weeks
		Change in language functions	Screening for Aphasia in Neurodegeneration (SAND)	
		Change in activities of daily living	ADCS-ADL score	
		Change in global cognition	MMSE	
		Change in GABA(B)ergic transmission	Long intracortical inhibition (LICI) Sort intracortical inhibition (SICI)	
		Prefrontal cortical oscillatory activity	TMS-EEG	
		Behavioral changes	NPI questionnaire	
		Global cognition changes	Addenbrooke’s Cognitive Examination Revised (ACE-R)	
		Behavioral functions	Frontal Behavioral Inventory	
**FTD_ET-STEM** NCT05315661	** Primary **	Dose-limiting toxicity (DLT)	Incidence rate of DLT	First 3-week cycle of treatment
		Safety and tolerability	AEs as assessed by CTCAE v5.0	Up to 5 years
	** Secondary **	Change in cognitive status	ADAS-Cog 13 response rate	Screening, after the first treatment, 12 weeks, 48 weeks, 96 weeks, 144 weeks, 192 weeks, 240 weeks
		Change in clinical assessment of dementia	CDR FTLD-SB score	
		Change in activities of daily living	ADCS-ADL score	
		Change in caregiver-administered (CGA) neuropsychiatric status	CGA-NPI Score	
		Change in mental status	Korean MMSE	
		Preliminary efficacy	CSF biomarkers	Up to 12 weeks
**Metformin for C9orf72 ALS/FTD** NCT04220021	** Primary **	Safety and tolerability of metformin	Number of subjects with treatment-emergent AEs	Baseline through 24 weeks
		Change in RAN protein levels	RAN protein levels in CSF	Baseline to week 24
	** Secondary **	Change in functional ability (capability and independence)	ALS Functional Rating Scale (ALSFRS-R) score	Baseline through week 52
**C9ORF72 ALS/FTD** NCT04993755	** Primary **	Safety and tolerability of TPN-101	Incidence and severity of treatment-emergent adverse events (TEAEs)	48 weeks
	** Secondary **	Pharmacokinetics of TPN-101	Concentrations of TPN-101 in plasma and CSF	48 weeks
		Pharmacodynamic effect of TPN-101 on neurodegeneration	CSF and blook NfL levels	
**SEL002** ACTRN = 12620000236998	** Primary **	Efficacy of Na_2_SeO_4_ as measured by change in global brain volume	MRI using SIENA and SIENAX—open-source imaging	52 weeks post-initiation of treatment
	** Secondary **	Safety and tolerability of sodium selenate	Frequency and severity of AEs, and the rate of study withdrawal Safety laboratory test 12-lead ECG Physical and neurological exam	At clinical visits and 52 weeks post-initiation of treatment
		Change in CSF tau levels	CSF total tau levels	Baseline and at week 52
		Cognitive function	Addenbrooke’s Cognitive Examination (ACE-III)	
		Behavior	Cambridge Behavioral Inventory (CBI-R)	

ADAS-Cog = Alzheimer’s Disease Assessment Scale-Cognitive subscale; ADCS-ADL = Alzheimer’s Disease Cooperative Study—Activities of Daily Living; AEs = adverse events; ARs = adverse reactions; AUC = Area Under the Curve; CDR = Clinical Dementia Rating; C-SSRS = Columbia-Suicide Severity Rating Scale; Cmax = maximum concentration; CTCAE = Common Terminology Criteria for Adverse Events; CSF = cerebrospinal fluid; FTLD = frontotemporal lobar degeneration; MRI = magnetic resonance imaging; NfL = neurofilament light chain; PGRN = Progranulin protein; MMSE = Mini-Mental State Examination; NACC FTLD-SB = National Alzheimer’s Coordinating Center Fronto-Temporal Lobar Degeneration Sum of Boxes; NPI = Neuropsychiatric Inventory; NIH EXAMINER = National Institutes of Health Executive Abilities Assessment; RAN = Ras-related nuclear; SAEs = serious adverse events; Tmax = time to maximum concentration; TMS-EEG = transcranial magnetic stimulation—electroencephalography.

## Data Availability

The original contributions presented in this study are included in the article/Supplementary Material. Further inquiries can be directed to the corresponding author.

## Supplementary Materials

The following supporting information can be downloaded at https://www.mdpi.com/article/10.3390/neurosci6040114/s1, File S1: PRISMA-ScR Checklist.

## Appendix A

**Table A1:** Search Strategy Details.

Clinical Trial Registry	Search Terms and Boolean Operators	Status and Age Filters Applied
National Library of Medicine Clinical Trials (ClinicalTrials.gov)	(“frontotemporal dementia” OR “FTD”) AND (“treatment” OR “disease modifying” OR “gene therapy”)	Status: Recruiting, Not yet recruiting, Active, Completed Age: Adults 18+years
European Union Clinical Trials Registry (EUCTR)	Frontotemporal dementia AND pharmacological	Status: Completed, Ongoing Age: Adults (18+ years)
Australian New Zealand Clinical Trials Registry (ANZCTR)	FTD OR Frontotemporal dementia	Status: Recruiting, Completed Age: Adults (18+ years)

## Appendix B

**Table A2:** Included Trials and Descriptive Quality Appraisal Matrix using the Oxford Quality Scoring System (Jadad Score).

Study Name	Randomisation	Blinding	Withdrawals	Total Jadad Score (0–5)	Quality Interpretation
upliFT-D	Non-randomised	Open-Label	Yes	1	Low Quality (Safety/Feasibility focus)
ASPIRE-FTD	Non-randomised	Open-Label	Yes	1	Low Quality (Safety/Feasibility focus)
PROCLAIM	Non-randomised	Open-Label	Yes	1	Low Quality (Safety/Feasibility focus)
FTD-GRN	Randomised	Double Blind	Yes	5	High Quality
INFRONT-3	Randomised	Double Blind	Yes	5	High Quality
SORT-IN-2	Non-randomised	Open-Label	Yes	1	Low Quality (Safety/Feasibility focus)
Veri-T-001	Randomised	Double Blind	Yes	5	High Quality
PEA-FTD	Randomised	Double Blind	Yes	5	High Quality
FTD_ET-STEM	Non-randomised	Open-Label	Yes	1	Low Quality (Safety/Feasibility focus)
Metformin for C9orf72 ALS/FTD	Non-randomised	Open-Label	Yes	1	Low Quality (Safety/Feasibility focus)
C9ORF72 ALS/FTD	Randomised	Double Blind	Yes	5	High Quality
SEL002	Randomised	Double Blind	Yes	5	High Quality
